# The effects of maternal flow on placental diffusion‐weighted MRI and intravoxel incoherent motion parameters

**DOI:** 10.1002/mrm.30379

**Published:** 2024-11-28

**Authors:** George Jack Hutchinson, Adam Blakey, Nia Jones, Lopa Leach, Neele Dellschaft, Paul Houston, Matthew Hubbard, Reuben O'Dea, Penny Anne Gowland

**Affiliations:** ^1^ Sir Peter Mansfield Imaging Centre, School of Physics and Astronomy The University of Nottingham Nottingham UK; ^2^ School of Mathematical Sciences The University of Nottingham Nottingham UK; ^3^ School of Medicine The University of Nottingham Nottingham UK; ^4^ School of Life Sciences The University of Nottingham Nottingham UK

**Keywords:** diffusion, IVIM, modelling, placenta

## Abstract

**Purpose:**

To investigate and explain observed features of the placental DWI signal in healthy and compromised pregnancies using a mathematical model of maternal blood flow.

**Methods:**

Thirteen healthy and nine compromised third trimester pregnancies underwent pulse gradient spin echo DWI MRI, with the results compared to MRI data simulated from a 2D mathematical model of maternal blood flow through the placenta. Both sets of data were fitted to an intravoxel incoherent motion (IVIM) model, and a rebound model (defined within text), which described voxels that did not decay monotonically. Both the in vivo and simulated placentas were split into regions of interest (ROIs) to analyze how the signal varies and how IVIM and rebounding parameters change across the placental width.

**Results:**

There was good agreement between the in vivo MRI data, and the data simulated from the mathematical model. Both sets of data included voxels showing a rebounding signal and voxels showing fast signal decay focused near the maternal side of the placenta. In vivo we found higher $f_{\text{IVIM}}$ in the uterine wall and near the maternal side of the placenta, with the slow diffusion coefficient D reduced in all ROIs in compromised pregnancy.

**Conclusion:**

A simulation based entirely on maternal blood explains key features observed in placental DWI, indicating the importance of maternal blood flow in interpreting placental MRI data, and providing potential new metrics for understanding changes in compromised placentas.

## 1 INTRODUCTION

DWI MRI is a valuable tool for investigating placental function across gestation,^1^, ^2^ is sensitive to different placental structures^3^ and can probe placental microstructure.^4^ It also varies in conditions such as preeclampsia (PE),^5^ fetal growth restriction (FGR),^6^, ^7^ and vascular malperfusion.^8^ The DWI signal is generally sensitive to any intravoxel movement (diffusion, perfusion, or flow) but it is not clear, which types of movement drive the DWI signal in the placenta.

Typically, DWI datasets are acquired at different values of diffusion weighting (*b* value) and are fitted to the intravoxel incoherent motion (IVIM) model^9^ for parameters conventionally associated with diffusion and perfusion within tissue. The diffusion component is usually attributed to restricted diffusion of water in the organ, and in most organs the perfusion component is assumed to reflect blood flow through capillary networks, which appears random on the spatial scale of a voxel and the time scale of the DWI encoding. However, as Le Bihan et al.^10^ pointed out in their original IVIM paper, larger scale movement of blood, for instance, in major vessels, can also cause intravoxel dephasing and hence, signal attenuation in this sequence,^11^, ^12^ and this effect may be expected in lakes of maternal blood within the placenta particularly close to the placental basal plate.

**FIGURE 1:**
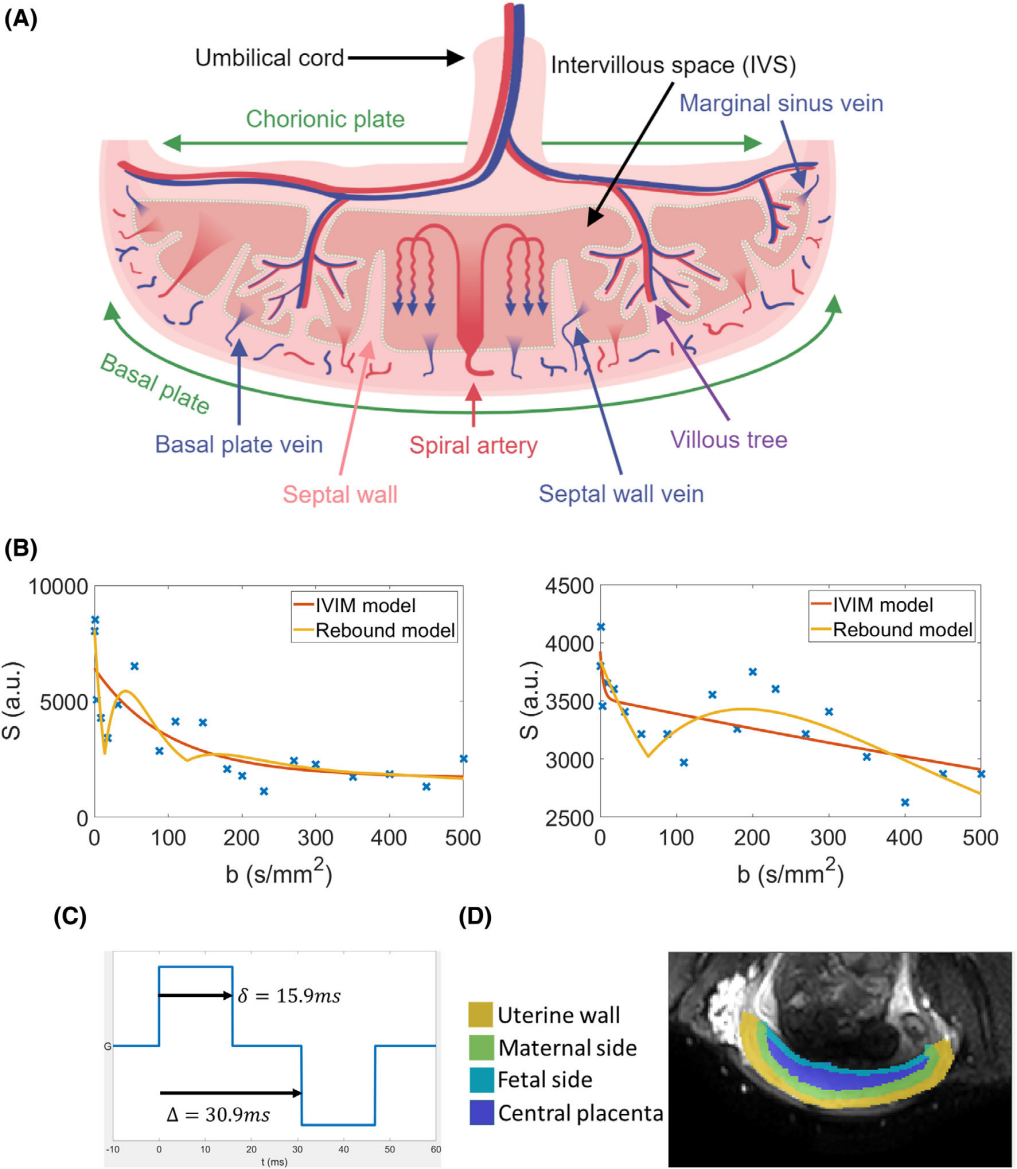
(A) Schematic illustrating the dual circulation of the placenta; a diagram of the simplified model used here is given in Figure S1. (B) Signal from two voxels plotted against *b*‐value, with the fits to the intravoxel incoherent motion (IVIM) and rebound models shown. (C) Timing of the encoding gradient in the pulse gradient spin echo (PGSE) pulse sequence, with the lobe length (δ) and diffusion time (Δ) labeled. (D) A *b* = 0 s/mm^2^ image with the regions of interest labeled.

In the placenta, the fetal blood flows within villous trees, which are bathed in maternal blood moving freely through the intervillous space (IVS) (shown in the placenta schematic Figure 1A). This maternal blood is supplied by spiral arteries, which are remodeled during healthy early pregnancies to provide a low resistance path for flow, transforming high velocity, high volume arterial blood flow into low velocity, high volume flow within the IVS. As the maternal blood enters the placenta it is generally driven into the central cavity of a placentone (region bounded by septa), from where it can percolate around the fetal villous trees, enabling the exchange of oxygen, nutrients, and waste products between the mother and fetus.^13^ Placental DWI is likely to be sensitive both to blood moving throughout the IVS and fetal blood within the villous trees. In compromised pregnancies (CP), incomplete spiral artery remodeling can lead to high resistance to flow, and higher velocity, lower volume flow into the placenta.

In this work, we simulated DWI MRI data using a mathematical model of maternal blood movement through the placenta to investigate the origins of features observed in placental DWI. In particular, we investigated cases where the DWI signal “rebounds” at certain *b*‐values (as illustrated in Figure 1B), rather than decaying monotonically as predicted by the standard IVIM model. We also investigated extremely rapid signal decays (here, termed ${\mathrm{Hi}}_{\text{IVIM}}$) often observed at the periphery of the placenta in DWI data.

The aim of this work was to use a mathematical model of the DWI signal expected from maternal blood flow through the human placenta to explain observed features of the placental DWI signal in healthy and CP. Our goal is a better understanding of in utero placental DWI data to develop novel biomarkers that can be used to identify CP.

## 2 METHODS

We first describe a mathematical model of blood flow across the placenta, and how it is used to simulate DWI signals. We, then, apply different analytic approaches to summarize and characterize the simulated DWI data and apply the same analytic approaches to in utero DWI data from compromised and healthy pregnancies (HP).

### 2.1 Mathematical blood flow model

We modeled the placenta as a rigid porous medium, with the resistance to maternal flow through the IVS corresponding to the presence of the fetal villous tree. For simplicity, we considered a time‐independent description in a 2D spatial domain representative of key structural features in the placenta. Briefly, we considered six placentones separated by septal walls, each containing one artery and one vein, with two larger marginal sinus veins included at the periphery of the domain. The widths of the arteries varied along their lengths as they entered the placenta, to capture spiral artery remodeling. Central cavity regions, in which there are no fetal villi, were included in the region near the artery mouth. Figure S1 shows a schematic of the computational domain considered with relevant dimensions given in Table S1.

Maternal blood flow velocity and pressure were described by the incompressible Navier–Stokes equations with an additional Darcy‐like resistance term, given by

(1) $$\Psi \;v-\mu \nabla^{2}v+\rho \left(v\cdot \nabla \right)v+\nabla p=0,$$

(2) ∇·v=0,

where v(x) is the unknown velocity, p(x) is the unknown pressure, $\rho =1\times 10^{4}\;\mathrm{kg}/m^{3}$ is the density, $\mu =4\times 10^{-3}\;\mathrm{Pa}\;s$ is the dynamic viscosity, and Ψ(x) is the effective resistance to flow through the fetal villous tree, which varies smoothly throughout the domain; we set $\Psi =\frac{\mu }{k}$ with $k=1\times 10^{-8}\;m^{2}$ (large flow resistance) in the IVS, and set Ψ=0 (no flow resistance) inside central cavitiesand vessels, with a small transition region in between (detail Data S1.).

Poiseuille flow with a peak speed of 0.35m/s (corresponding to peak systole inflow of 0.1m/s at the artery mouth)^13^ was specified on the six arterial inflows, free‐flow boundary conditions were imposed on the vein exits, and no‐slip elsewhere on the boundary.

We numerically solved Eqs. (1) and (2) using discontinuous Galerkin finite element methods,^14^, ^15^ implemented in AptoFEM.^16^ Mesh generation was undertaken using Gmsh,^17^ with refinement close to arteries, veins, and the central cavity transition region; the resulting mesh comprised 158 784 triangular elements. See Cockburn et al.^18^ for full details of the numerical method used.

### 2.2 Simulated MRI signal

A 2D grid of 91 × 19 pixels, of size 2.5 × 2.5 mm^2^ (corresponding to the resolution of the in vivo MRI scans) was arranged over the computational domain described above. Within each pixel we defined a uniform grid of 20 × 20 points corresponding to the initial positions $x^{j}\left(0\right)$ for each of N=400 isochromats located in the pixel. By sampling the velocity field computed as described in Section 2.1 at each of these points, we simulated the movement of these isochromats during DWI encoding assuming that the velocity of each isochromat $v^{j}$ remained constant during the encoding period from t=0 to δ+Δ where δ is the encoding gradient lobe length, and Δ is the diffusion time in the pulsed gradient spin echo (PGSE) sequence used experimentally (Figure 1C). We calculated the resulting phase change of each (j) isochromat in the PGSE sequence for each (k) gradient amplitude using.

(3) $$\phi^{j,k}=\gamma \int_{0}^{\delta +\Delta }G^{k}\left(t\right)\cdot \left(x^{j}\left(t\right)-x^{j}\left(0\right)\right)\;dt,$$

where $G^{k}\left(t\right)$ is the magnetic field gradient amplitude corresponding to the kth *b*‐value, and $x^{j}\left(t\right)$ is the position of the jth isochromat at time t (determined from the sampled velocity $v^{j}$) with initial position $x^{j}\left(0\right)$, and γ is the gyromagnetic ratio. The resulting signal from each pixel at each *b*‐value was calculated by summing the complex signal over all isochromats initially located within the pixel assuming equal transverse magnetization existed in each isochromat,

(4) $$S^{k}=\left|\sum_{j=1}^{N}\exp\left(-i\phi^{j,k}\right)\right|.$$

This was calculated for the gradient aligned along each axis of the 2D grid in turn and the two resulting magnitude signals were averaged. This was then repeated for 19 different gradient amplitudes used in vivo (Table 1). Gaussian‐distributed noise (mean zero and SD of 1% of the maximum signal $S_{0}$) was then added to each of the resulting signals $S^{k}$, matching the approximate level of noise found in vivo.

**TABLE 1:** Table of scan parameters used.

Voxel size	2.5 × 2.5 × 6 mm^3^
FOV	400 × 400 × 66 mm^3^
Lobe length (δ)	15.9 ms
Diffusion time (Δ)	30.9 ms
TE	61.4 ms
TR (minimum)	1800 ms
*b* values	0, 1, 3, 9, 18, 32, 88, 110, 147, 180, 200, 230, 270, 300, 350, 400, 450, 500 s/mm^2^
NSL	3–6

### 2.3 In vivo data

Twenty‐two participants were scanned with local ethics approval. They were 18 to 45 years old, with body mass index <30 kg/m^2^ and singleton pregnancy between 28 and 36 week's gestational age; 13 were considered to have HP, and nine had CP (1 PE, 3 fetal growth restriction [FGR], 4 PE and FGR, 1 PE and anemia). PE was defined as blood pressure >140/90 mm Hg on two separate occasions >4 h apart, and proteinuria of >30 mmg/mmol on protein creatine ratio or 300 mg in a 24 h urine collection. FGR was defined as women expecting a growth restricted baby, below the 10th centile for birth weight. This data has been published previously^19^ where it was fitted to a simpler IVIM model. In vivo imaging was carried out using respiratory gated PGSE‐EPI (parameters given in Table 1) on a Philips 3 T Ingenia system in normal operating mode with whole body averaged specific absorption rate <2.0 W/kg. Data were collected using posterior receive arrays that were built into the scanner bed, combined with an anterior abdominal array and participants were positioned at approximately 30° tilt to prevent vena cava compression.

Volumes were inspected and those showing >5 mm displacement from a placental mask drawn on the *b* = 0 s/mm^2^ volume were discarded from analysis, (33/418 volumes discarded, Table S2). The data was split into four regions of interest (ROIs) (Figure 1D). The uterine wall adjacent to the placenta was manually segmented on the *b* = 0 s/mm^2^ images to create an ROI to investigate the spiral arteries and vasculature within the uterine wall. The placenta was then segmented and split into three ROIs; maternal side (including basal plate, voxels within 10 mm of maternal margin of placenta), fetal side (including chorionic plate, within 5 mm of the fetal margin of placenta), and center (remaining voxels). Overlap between the maternal and fetal side was classed as maternal side and slices where the placenta was <5 voxels across, or where the geometry of the placenta meant it could not be split into the three ROIs were discarded from analysis. This process was semi‐automated to simplify the selection of ROIs.

### 2.4 Analysis of in vivo and simulated MRI data

The in simulated and in vivo signals were both fitted pixel/voxelwise to two related models, the standard IVIM model and a novel rebound model, and fitted parameters were compared over the four ROIs defined above. To fit to the standard IVIM model

(5) $$S\left(b\right)=S_{0}\left[\left(1-f_{\text{IVIM}}\right)e^{-\mathrm{bD}}+f_{\text{IVIM}}e^{-bD^{*}}\right],$$

first the low *b*‐value (*b* ≤ 110 s/mm^2^) data and high *b*‐value (*b* ≥ 200 s/mm^2^) data were fitted to mono‐exponential decays to provide estimates of $D^{*}$ and D, which were then used as initial guesses in a fit to the full IVIM model (Eq. [5]). Parameters were constrained in the following ranges: D: 0–25 × 10^−3^ mm^2^/s, $D^{*}$: 0–1000 × 10^−3^ mm^2^/s, and $f_{\text{IVIM}}$: 0–1.

In both the simulated and in vivo data, it was observed that in some pixels/voxels the signal did not decay monotonically as expected from the IVIM model (Eq. [5]). To identify these voxels we created a phenomenological rebound model

(6) $$S\left(b\right)=S_{0}\left[\left(1-f^{\prime }\right)e^{-bD_{1}}+f^{\prime }|\cos\left({\mathrm{cv}}^{\prime }\right)|e^{-bD_{2}}\right],$$

where $v^{\prime }$ is a free parameter to be fitted, $c=\gamma m_{1}$ (units sm^−1^) and $m_{1}$ is the first moment of the gradient, that is, c=γGδΔ assuming square rather than trapezoidal gradient lobes and is closely related to √b, (below we use the term b‐value when discussing both flow and apparent diffusion effects for clarity). This model was motivated by Le Bihan et al.^10^ who determined that the attenuation factor for plug flow in isotopically distributed capillaries should be a sinc function attenuated by the bulk diffusion coefficient ($D_{1}$). However, we generalized Le Bihan's model to capture the range of patterns of flow exhibited in the placenta, by replacing the sinc function with a cosine attenuated by the fast apparent diffusion coefficient ($D_{2}$), to capture potentially larger rebounds (as observed), and attenuating the rebound by $D_{2}$ (rather than the diffusion coefficient $D_{1}$) to recognize that other dephasing processes may be occurring link to the rebound in the voxel. The amplitude and *b*‐value of occurrence of the rebound depends on $m_{1}$ and $v^{\prime }$ (that relates to the velocity of the isochromats within the voxel).

To fit data to the rebound model, $v^{\prime }$ was constrained between 0 and 0.0025 and three initial guesses of $v^{\prime }$ (0, 0.001 and 0.002) were considered to address local minima, with the fit that gave the smallest sum of squares being taken as the final optimal solution. For in utero data, an *F* test was used to compare the rebounding and IVIM fits, and the fraction of voxels/pixels where the rebounding model showed a significant improvement over the IVIM model was noted.

Regions of very fast signal decay ($D^{*}>100\times D$) were observed in both in vivo and simulated data. We defined voxels as being high IVIM (${\mathrm{Hi}}_{\text{IVIM}}$) if $f_{\text{IVIM}}$ >0.5 and $D^{*}$ >300 × 10^−3^ mm^2^/s; the fraction of ${\mathrm{Hi}}_{\text{IVIM}}$ voxels/pixels was calculated for each of the four ROIs.

For the HPs, results for each measure in each ROI were compared to the central ROI in turn (2‐tail paired *t* test, false discovery rate [FDR] corrected across 15 tests). Each measure was also compared between HP and CP for each ROI (1‐tailed unpaired *t* test, FDR corrected across 20 tests). ${\mathrm{Hi}}_{\text{IVIM}}$ is a derived parameter but the significance threshold was the same when it was excluded.

## RESULTS

**FIGURE 2:**
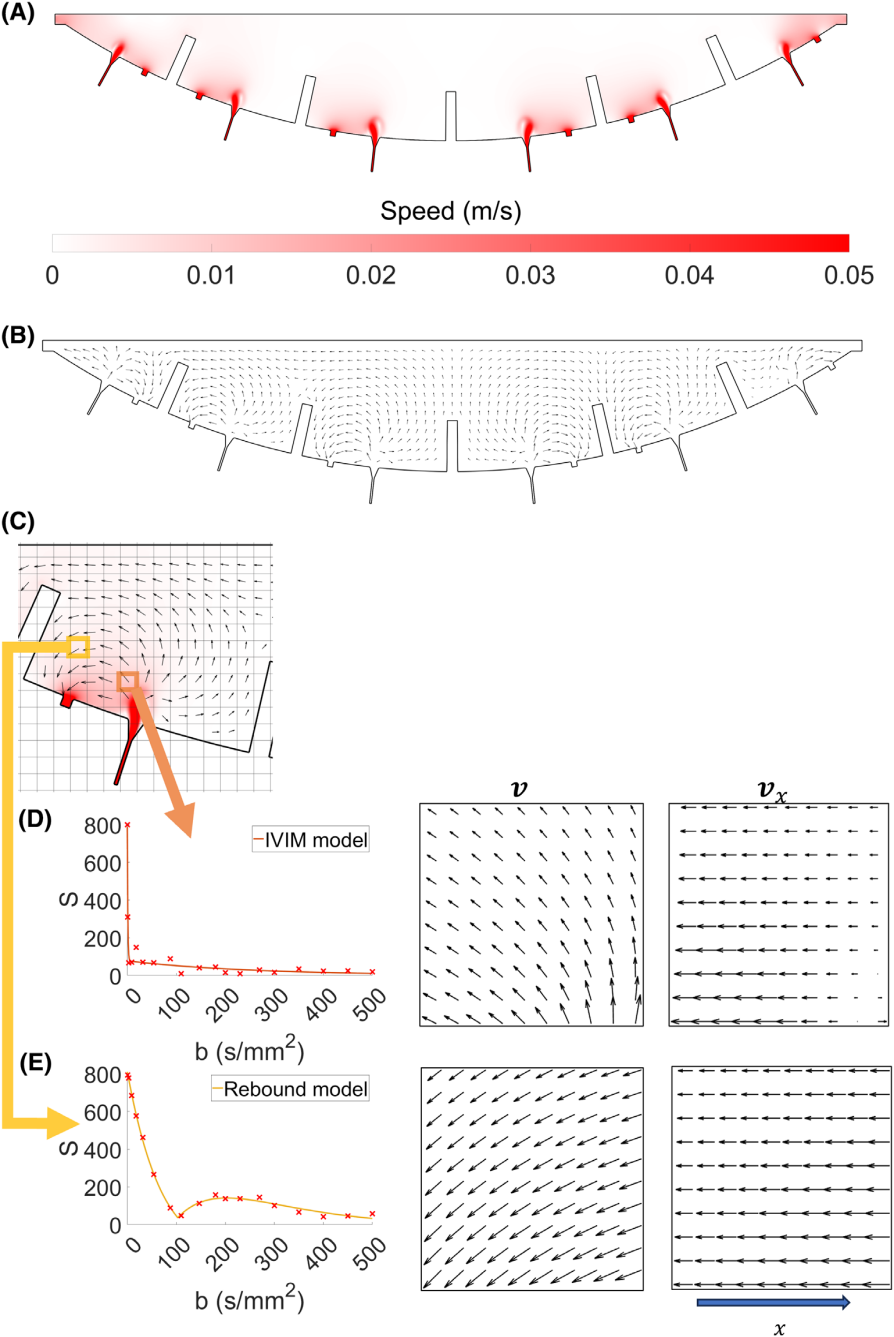
The simulated data for maternal blood flow through the placenta and the corresponding maps produced from the intravoxel incoherent motion (IVIM) and rebound fits of this data. (A) Shows the speed of blood (maximum velocity 0.35 m/s but thresholded to 0.05 m/s for better visualization), and (B) shows a quiver plot of the velocities sampled at each isochromat, where the length of each arrow is scaled by $\log\left(\left|v\right|/10^{-5}\right)$. (C) A zoomed in view of a single placentone, with the colors and arrows taken from (A,B). (D,E) The velocity of individual isochromats in two voxels, the simulated signal decays for these voxels in an x‐gradient and fit to the data, and also the x‐component of the velocities.

Figure 2A shows the simulated speed of maternal placental blood flow ∣v∣, and Figure 2B indicates velocity, with the length of each arrow determined by the logarithmically scaled mean speed within each pixel (direction only shown in Figure S2A). Figure 2C shows simulated maternal flow across a single placentone in more detail. Resistance to flow is shown in Figure S2B.

Figure 2D,E highlight the velocities of the isochromats within two pixels with the corresponding and signal decay plots. In Figure 2D, the rebound model fit shown did not outperform the IVIM model fit for that pixel, whereas in Figure 2E the IVIM model fit shown was outperformed by the rebound model. In Figure 2E, the net velocity (v) is bending toward an outlet, producing a gradual variation in $v_{x}$ across y. Depending on the first gradient moment of the sequence the phase of spins with higher $v_{x}$ will wrap and rephase with those of lower $v_{x}$ causing a rebound at *b*‐value ˜200 s/mm^2^. In Figure 2D, however, the variation in $v_{x}$ along x changes rapidly across the y direction, meaning although the faster spins do rephase, they dephase again rapidly causing rapid signal loss and a pixel characterized by $Hi_{\text{IVIM}}$. Given a high enough sample rate of *b*‐values it is likely these pixels would show a small, quickly oscillating rebound, which in vivo would be below the noise floor.

**FIGURE 3:**
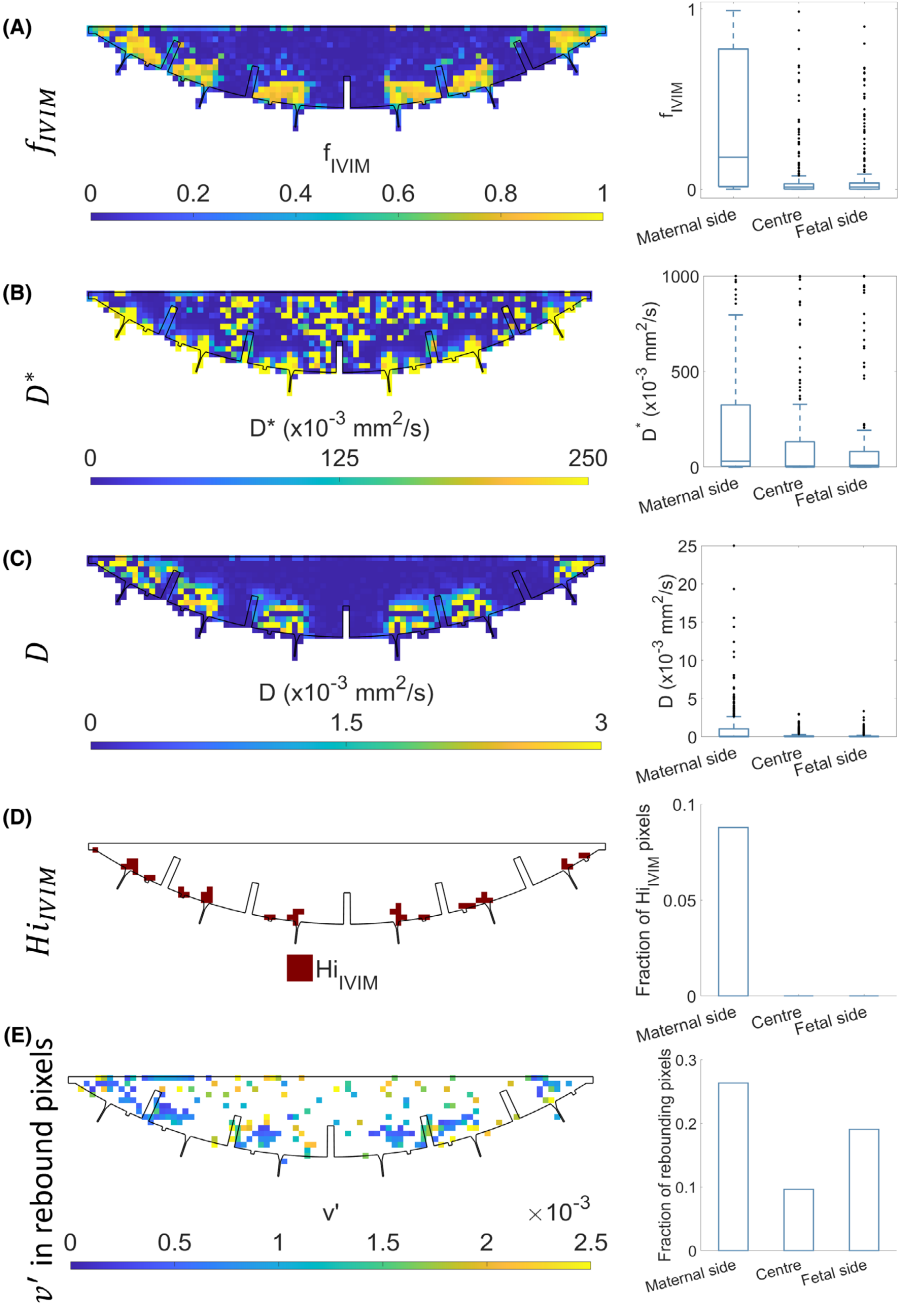
Maps of intravoxel incoherent motion (IVIM) model fitted to the simulated data for maternal blood flow through the placenta. (A–C) Contains box plots showing results averaged over maternal side, central, and fetal regions of interest, and (D,E) show bar charts counting the fraction of pixels in each.

Figure 3A–E shows parameter maps for the IVIM fit to the simulated data with bar charts showing the distribution of parameter values across similar ROIs to those drawn in vivo (the uterine wall was not simulated). Figure 3E maps $v^{\prime }$ for the 19% of pixels where the rebound model significantly outperformed the IVIM model and shows that these occurred mostly in the maternal ROI close to the inlets and outlets, but also somewhat on the fetal ROI. The full map for $v^{\prime }$ is shown in Figure S2C. The random noise that was added to the simulation caused small local maxima on the decay curves, which were detected by the rebound fit even if no significant peak was present, emphasizing the need to use an *f*‐test to determine when the rebound fit is meaningful.

**FIGURE 4:**
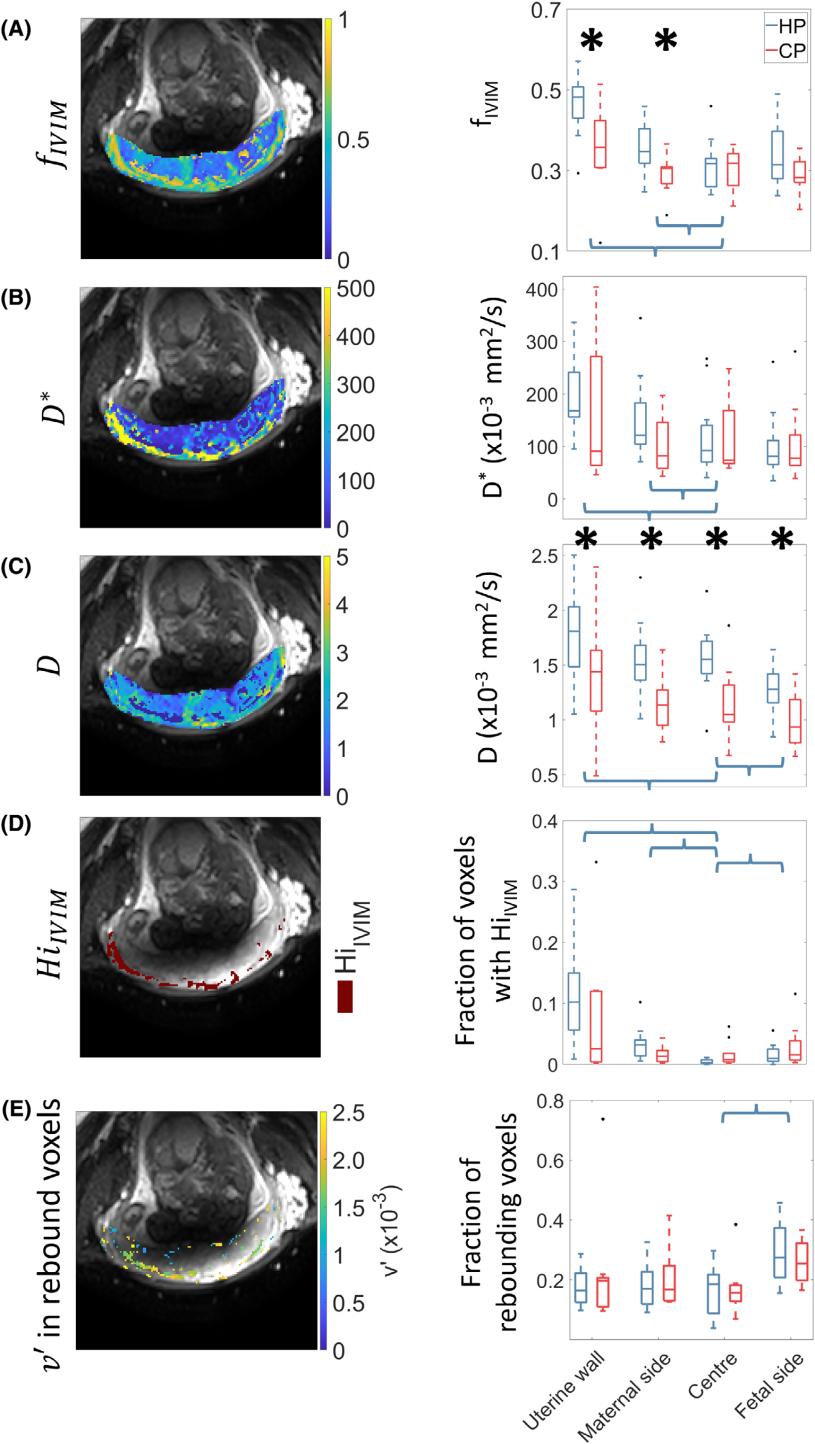
Parameter maps for fits to the data from a healthy pregnancy (HP03) and box plots showing results of the fits in each regions of interest averaged across healthy pregnancies (HP) (blue) and compromised pregnancies (CP) (red) subjects (group mean with SD, individual subject data is shown in Figure S5). *Indicates significant difference between HP and CP (1‐tailed unpaired *t* test, FDR corrected across 20 tests) and bracket indicates significant difference between ROI and central ROI in HP (2‐tail paired *t* test, false discovery rate [FDR] corrected across 15 tests).

Figure 4 shows an example of $f_{\text{IVIM}}$, $D^{*}$, and D parameter maps from the IVIM fit, voxels defined as ${\mathrm{Hi}}_{\text{IVIM}}$, and $v^{\prime }$ parameter map from the rebound model fit, masked for voxels where the rebound model significantly outperformed the IVIM model (the corresponding unmasked map is shown in Figure S3). Additional examples for a further healthy pregnancy and compromised pregnancy are shown in Figure S4. The box plots in Figure 4 summarize the results in each of the four ROIs, averaged over all subjects for the control and compromised pregnancy groups separately; individual subject data is shown in Figure S5.

Considering HP (HP‐Table 2), $f_{\text{IVIM}}$ and $D^{*}$ were both significantly higher in the uterine wall and maternal side ROIs than in the central ROI. The derived ${\mathrm{Hi}}_{\text{IVIM}}$ parameter was significantly larger in the uterine wall, maternal side as well as the fetal ROIs compared to the central ROI. The rebound fraction was only higher in the fetal side ROI compared to the central ROI and D was higher in the uterine wall ROI and lower in the fetal ROI compared to the central ROI.

**TABLE 2:** Statistically significant differences in IVIM measures between each ROI and the central ROI in HP.

	Uterine wall vs. center	Maternal region vs. center	Fetal region vs. center
$f_{\text{IVIM}}$	*p* = 6 × 10^−6^	*p* = 0.02	
D	*p* = 0.001	*p* = 0.006	
${\mathrm{Hi}}_{\text{IVIM}}$ (derived from $f_{\text{IVIM}}$ and 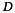 )	*p* = 0.0003	*p* = 0.0009	*p* = 0.009
D	*p* = 0.014		*p* = (4 × 10^−6^)
Rebound fraction			*p* = 2 × 10^−6^

*Note*: In every case, the value was greater except when the number is in parentheses.

Abbreviations: HP, healthy pregnancies; IVIM, intravoxel incoherent motion; ROI, region of interest.

Comparing HP and CP, the CPs had significantly lower $f_{\text{IVIM}}$ in the uterine wall and maternal side ROIs and significant lower D in all ROIs (Table 3).

**TABLE 3:** Statistical significance of decreases observed in CPs compared to HPs.

	Uterine wall	Maternal ROI	Central ROI	Fetal ROI
$f_{\text{IVIM}}$	Lower *p* = 0.005	Lower *p* = 0.009		
$D^{*}$				
${\mathrm{Hi}}_{\text{IVIM}}$ (derived from $f_{\text{IVIM}}$ and $D^{*}$)				
D		Lower *p* = 0.003	Lower *p* = 0.003	Lower *p* = 0.009
Rebound fraction				

Abbreviations: CP, compromised pregnancies; HP, healthy control pregnancies; IVIM, intravoxel incoherent motion; ROI, region of interest.

## 3 DISCUSSION

Here, we have used a mathematical model of flow through the IVS to explain features of placental in utero DWI data not observed in other tissues and to support the development of efficient markers of altered placental flow in compromised pregnancy.

### 3.1 Rebounds and rapid decay

The original IVIM model described the effects of capillary flow in tissue, assuming that this could be modeled as a random walk at the length scale of the MRI voxel and time scale of DWI encoding.^10^ The placenta contains a large volume fraction of maternal blood moving within the IVS, unconstrained by a vascular network but impeded by the villous trees, and in this case the standard biexponential IVIM model cannot describe all observed behavior. We found that for many, frequently clustered, voxels in the placenta, the signal did not decay monotonically with b‐value, but instead exhibited rebounds superimposed on a signal decay. In some other voxels, we observed extremely fast signal decay, ${\mathrm{Hi}}_{\text{IVIM}}$, which is not found in tissues with more typical blood volume fractions.^9^ Similar effects were observed in simulations (Figure 2D,E).

The concept of moving spins being rephased has long been understood in MRI for both bulk flow^20^ and tissue perfusion,^21^ although we are not aware of this ever having previously been reported in vivo, probably because of the small blood volume fraction in most tissues. We had previously observed rebounds in the placenta (e.g., Figure 3 in Dellschaft et al.^19^), but had overlooked their relevance, and rebounds observed in voxelwise time courses will generally dissipate when the signal is averaged across a larger ROI.

To detect and characterize the rebound signal we developed a phenomenological rebound model (Eq. [6]), which was motivated by Le Bihan's model for plug flow in isotopically orientated capillaries, but modified to allow larger rebounds and decoupling of their amplitude from the gradient moment at which they occur (b‐value in Eq. [6]).

In our model, the rebound was damped by $D_{2}$, which characterizes the effects of all movement causing the spins to lose coherence on the time and length scale of PGSE encoding, whereas Le Bihan's model for plug flow damped the rebound by D corresponding to diffusion in tissue. When we applied this model to simulated data (Figure 3) we found that pixels displaying rebounds were likely to be found close to the inlets and outlets from the placenta. In vivo (Figure 4, Figure S4, and Table 2) we found most rebounds in the fetal side ROI, probably because of blood changing direction as it reaches the chorionic plate (as seen in the simulation). This difference is discussed further below.

The simulations showed that ${\mathrm{Hi}}_{\text{IVIM}}$ resulted from the complex patterns of movement in regions of the IVS where there is rapid blood flow (Figure 2E), and again, this often occurs close to spiral artery inlets and venous outlets (Figure 3). ${\mathrm{Hi}}_{\text{IVIM}}$ was also found close to the maternal wall in utero (Figure 4). It is possible that if we had looked in more detail at low b‐values we might have seen rebounds in some ${\mathrm{Hi}}_{\text{IVIM}}$ pixels; indeed small rebounds can be seen in the simulated signal decay in Figure 2E, although these would be too small to be detected in utero. This shows that many different flow patterns can give rise to similar effects.

### 3.2 Simulated data

The simulation of flow in the IVS was deliberately simple, modeling percolation through the villous trees as resistance to flow, with no inclusion of fetal villous flow, percolation through the villous trees or diffusion within blood or tissue. This allowed us to isolate the effect of bulk IVS flow on the MRI DWI signal. Importantly, this simple model was able to predict both rebounds and very rapid IVIM signal decays (${\mathrm{Hi}}_{\text{IVIM}}$), generally occurring close to the periphery of the placenta (Figure 2).

Figure 2 indicates that it is unlikely that a single model could fully characterize the flow within the IVS, particularly once percolation through the villous trees is included. However, some general conclusions can be reached because generally dephasing occurs in the PGSE sequence for flow fields with a nonzero curl (considering the component of flow in the direction of the gradient), but rephasing can occur in various situations. For instance, if a pixel contains two compartments, each with a distinct velocity then full rephasing will occur at a particular b‐value (neglecting diffusion). Alternatively, if there is a constant shear across the pixel in one or more dimensions, then the signal response to linearly increasing gradient amplitude (˜√b) will be in the form of a sinc function.

The simulation predicts that blood entering the base of the spiral artery at 35 cm/s decelerates to ˜10 cm/s near the spiral artery mouth,^13^ subsequently decelerates as it passes through the IVS (because of dissipation of spiral artery inflow across the whole of the IVS and resistance to flow), and finally accelerates again as it exits the IVS through basal plate veins or the marginal sinus. In this 2D model, in which all veins are assumed to be in the basal plate (none on the septa), most blood enters and leaves within the same placentone (˜97% in central placentones). Ongoing work is considering the effect of the positions of the veins on the flow in the placenta.

The model predicts that most blood in the IVS is moving very slowly, leading to the slow signal decays in the central ROI (Figures 2 and 3) in agreement with in utero data (Figure 4). The mean blood speed over the entire placenta (including IVS, villi, and villous vessels) was ˜0.29 cm/s, (the slowest blood flow was ˜0.01 cm/s found in the IVS near the chorionic plate). A previous computational model predicted a mean speed between 0.3 and 6 cm/s over a 2D placentone, depending on the inlet speed at the artery mouth.^22^ A further study estimated a mean blood speed of 0.06 cm/s^23^ based on bulk flow averaged over the placental volume and historical radioactive dye data. The variation in predictions from the different approaches is likely to be because of variations in assumptions related to inlet speed, fraction of space occupied by the IVS, and outlet locations.

However, the mean speeds predicted by our model agree with our previous experimental measurements of less than 0.2 cm/s in the healthy placenta using phase contrast angiography^19^ and supports long transit times measured by others using contrast‐enhanced MRI^22^ and arterial spin labelling MRI.^24^

The simulated data (Figure 3C) shows decay consistent with the diffusion term D, particularly around inlet and outlet sites, despite no Brownian motion being simulated. This indicates that maternal flow can mimic slow diffusive components, and that in IVIM analyses D is likely to be influenced in part by bulk flow.

### 3.3 In utero comparison between ROIs and groups

In HP, the new analysis found that $f_{\text{IVIM}}$ and $D^{*}$ were greater in the uterine and maternal ROIs compared to the central ROI, indicating the effect of flow through and from the spiral arteries into the IVS. A similar result was found for ${\mathrm{Hi}}_{\text{IVIM}}$, as expected given that it is derived from $f_{\text{IVIM}}$ to $D^{*}$.

The larger rebound fraction found in utero on the fetal side was not observed in the simulation (which found larger rebound on the maternal side), but none the less the simulation (Figure 2) suggest a mechanism for this rebound could be the change in direction of the maternal blood as it hits the chorionic plate and then returns to the venous outlets in the basal plate, septa, and at the peripheral sinus. The difference in simulated and in utero suggests that simulated flow was slower on the fetal side than in reality. We suspect that this is partly because we have assumed venous return through the basal plate and marginal sinus. Venous return is a generally neglected factor in placental function, and we are currently undertaking in utero and ex utero studies to elucidate all venous return pathways in the placenta. The simulations also neglected fetal blood flow, which may cause additional dephasing that could damp rebounds. Interestingly, in utero D was higher in the uterine wall and lower in the fetal ROI compared to the central ROI probably reflecting to the effects of bulk flow as well as different tissue fractions and different length scales of movement between and within the fetal villous tree in these regions.

In vivo $v^{\prime }$ maps show clustered regions of similar values of $v^{\prime }$ across the placenta (Figure 4), and the *F*‐test showed that the rebound model better described the data than the IVIM model in regions generally close to the uterine wall, further supporting the idea that these rebounds reflect local flow.

In CP, $f_{\text{IVIM}}$ was lower in the uterine wall and on the maternal side of the placenta, which is consistent with the expected effects of improper spiral artery remodeling causing slower flow in the uterine wall (because of higher downstream resistance), but faster jets of blood into the placenta, which would lead to smaller pockets of very high IVIM surrounded by areas with less movement (increased villous density might produce similar effects in principle). Further work is needed to investigate whether $Hi_{\text{IVIM}}$ in a region close to the basal plate might provide a marker of pregnancy compromise.

If the rebounds are caused by the maternal blood flow then we might expect the number to be reduced in placental disorders such as preeclampsia. No change was seen in the fraction of rebounding voxels in the CP, but further work is required using a more complete simulation to better understand the most relevant metrics to characterize the rebounds.

*D* was lower in the maternal, central and fetal ROIs in compromised compared to HP, suggesting altered small scale movements potentially reflecting altered percolation and flow through the villous trees as expected in CP.

These trends were similar to those previously reported for the same data, but with a different fitting approach.^19^ Previously a Kurtosis model was used, with D limited from 0 to 10 × 10^−3^ mm^2^/s and $D^{*}$ from 0 to 5000 × 10^−3^ mm^2^/s. In this analysis, D was allowed to fit across a wider range of values (similar to Slator et al.^25^) and a smaller range for $D^{*}$ as we could not reasonably measure larger values of $D^{*}$ with the b‐values selected. The previous analysis^19^ did not find lower $f_{\text{IVIM}}$ in the uterine wall of CP, but did show reduced $f_{\text{IVIM}}$ in the basal plate, which was largely included in the uterine wall ROI this time. Considering HP, a significant difference was found between the chorionic plate and placenta previously but was not between the larger fetal ROI and placenta ROI this time.

### 3.4 Limitations and further work

In this work, the ROIs were created automatically with a view to simple future translation. However, a comparison of these results to those from the previous analysis (that used different ROIs)^19^ and the simulation results suggests that future work should formally investigate the choice of ROIs used to characterize placental function, in particular focusing on the basal plate (location of the spiral arteries) and chorionic plate.

This work used a simple mathematical model of flow of maternal blood through the IVS to predict key features of in utero placental DWI data. It is likely that the MRI signal is dominated by maternal blood, which we estimate makes up 60% of the placental volume in utero (based on estimated villous volume fraction^26^ and change in placental volume on delivery^27^). A more complete model including percolation between the villous trees and flow through the fetal capillaries may reduce differences between the simulated and in utero data close to the chorionic plate (Figures 3 and 4), and will allow us to identify features of the DWI signal that can best highlight the effects of changes in villous structure in CP.^28^ Such a model is now being developed and will also include oxygen transfer, to identify joint features of DWI and MRI oxygenation measures that might be clinically relevant.

Previously, we have fitted this in vivo data using a Kurtosis model to characterize non‐Gaussian diffusion within the IVS. However, the Kurtosis model cannot describe a rebound and was excluded here to avoid overfitting. The difference in D observed in utero between CP and HP in all ROIs in this work, may have been caused by changes in IVS percolation or changes in tissue microstructure. Percolation is most likely to dominate in the central regions of the placenta, whereas bulk IVS flow (causing rebounds) is likely to be most relevant close to the basal plate and chorionic plate. In future, to avoid over fitting data different fits might be applied in different ROIs. There are also additional approaches that could be taken to the final analysis of the data, for instance, a joint histogram approach, or even the calculation of the product of $f_{\text{IVIM}}$ and $D^{*}$ may be more useful than ${\mathrm{Hi}}_{\text{IVIM}}$, (high $f_{\text{IVIM}}$ and $D^{*}$).

The simulations show that many different flow patterns may give rise to similar effects in the PGSE sequence used here; it is likely that it will be possible to overcome this by exploiting the additional information available from the sequence. For instance, using a sequence with different levels of flow compensation or different encoding lengths (∆) will provide varying sensitivity to velocity and acceleration. Furthermore, the magnitude of the signal in the PGSE sequence is determined by the range of velocities in the voxel, whereas the phase of the signal depends on the net velocity in the voxel, which can be measured at low b‐values (where flow encoding venc is low). Our simulation provides a method of optimizing pulse sequences for separating different types of movement before attempting to acquire in utero data.

The PGSE sequence is highly sensitive to motion and so any movement of the placenta, including movement because of contractions, could cause variations in the signal in this sequence. This is unlikely to cause the coherent pattern of rebounds, but would certainly perturb the fit. However, such movement is likely to be large scale across the placenta rather than local (as seen in rebounds). We excluded images that showed too much movement, but future work should consider additional methods of registration, detecting corrupted data and oversampling. We have developed a method of monitoring dynamic changes in velocity within the placenta to investigate this further.^29^

## 4 CONCLUSION

We have shown that key features of the placental DWI signal can be explained by a simple mathematical model of maternal blood flow through the IVS. This provides a better mechanistic understanding of placental DWI data and suggests metrics for detecting relevant changes in compromised pregnancy.

## FUNDING INFORMATION

Wellcome Leap In Utero Programme (SWIRL); National Institutes of Health (Pipox); funded through a studentship from Oxford‐Nottingham Biomedical Imaging Medical Research Council/Engineering and Physical Sciences Research Council (EPSRC) to G.H; funded through a studentship from EPSRC to A.B.

## Supporting information


**Figure S1.** Figure of simulated placenta and placentone geometry.
**Figure S2.** Figure of resistance of flow, flow direction and v′ for the simulated placenta.
**Figure S3.** Figure unmasked v′ fit, showing all placental voxels.
**Figure S4.** Figure showing additional example maps for a further healthy pregnancy and a compromised pregnancy.
**Figure S5.** Figure showing individual subject data from the fIVIM parameter boxplots in Figure 4.
**Table S1.** Table of dimensions of placental geometry and parameters associated with the blood flow model.
**Table S2.** Table of discarded volumes per participant.
**Data S1.** Detail of placental transition region.

## Data Availability

Data are available from the Sir Peter Mansfield Data Access Committee for researchers who meet the criteria for access to confidential data.

## References

[1] Moore RJ , Strachan BK , Tyler DJ , et al. In utero perfusing fraction maps in normal and growth restricted pregnancy measured using IVIM echo‐planar MRI. Placenta. 2000;21:726‐732. doi:10.1053/plac.2000.0567 10985977

[2] Capuani S , Guerreri M , Antonelli A , et al. Diffusion and perfusion quantified by magnetic resonance imaging are markers of human placenta development in normal pregnancy. Placenta. 2017;58:33‐39. doi:10.1016/j.placenta.2017.08.003 28962693

[3] You W , Andescavage N , Zun Z , Limperopoulos C . Semi‐automatic segmentation of the placenta into fetal and maternal compartments using intravoxel incoherent motion MRI. Proc SPIE Int Soc Opt Eng. 2017;10137:1013726. doi:10.1117/12.2254610 28947842 PMC5609721

[4] Slator PJ , Hutter J , McCabe L , et al. Placenta microstructure and microcirculation imaging with diffusion MRI. Magn Reson Med. 2018;80:756‐766. doi:10.1002/mrm.27036 29230859 PMC5947291

[5] Moore RJ , Stephen OS , Damian TJ , et al. Spiral artery blood volume in normal pregnancies and those compromised by pre‐eclampsia. Neuroimage. 2008;21:376‐380. doi:10.1002/nbm.1199 17893947

[6] Liu XL , Feng J , Huang CT , Mei YJ , Xu YK . Use of intravoxel incoherent motion MRI to assess placental perfusion in normal and fetal growth restricted pregnancies on their third trimester. Placenta. 2022;118:10‐15. doi:10.1016/J.PLACENTA.2021.12.019 34995915

[7] Sohlberg S , Mulic‐Lutvica A , Olovsson M , et al. Magnetic resonance imaging‐estimated placental perfusion in fetal growth assessment. Ultrasound Obstet Gynecol. 2015;46:700‐705. doi:10.1002/uog.14786 25640054 PMC5063104

[8] Kristi BA , Ditte NH , Caroline H , et al. Placental diffusion‐weighted MRI in normal pregnancies and those complicated by placental dysfunction due to vascular malperfusion. Placenta. 2020;91:52‐58. doi:10.1016/j.placenta.2020.01.009 32174307

[9] Le Bihan D . What can we see with IVIM MRI? Neuroimage. 2019;187:56‐67. 29277647 10.1016/j.neuroimage.2017.12.062

[10] Le Bihan D , Breton E , Lallemand D , Aubin ML , Vignaud J , Laval‐Jeantet M . Separation of diffusion and perfusion in intravoxel incoherent motion MR imaging. Radiology. 1988;168:497‐505. doi:10.1148/radiology.168.2.3393671 3393671

[11] Wetscherek A , Stieltjes B , Laun FB . Flow‐compensated intravoxel incoherent motion diffusion imaging. Magn Reson Med. 2015;74:410‐419. doi:10.1002/mrm.25410 25116325

[12] Anblagan D , Deshpande R , Costigan C , et al. Measuring coherent blood flow in the placenta, basal plate and chorionic plate. Proceedings of the 19th Annual Meeting of ISMRM, Montreal, Canada. The International Society for Magnetic Resonance in Medicine; 2011.

[13] Burton GJ , Woods AW , Jauniaux E , Kingdom JCP . Rheological and physiological consequences of conversion of the maternal spiral arteries for uteroplacental blood flow during human pregnancy. Placenta. 2009;30:473‐482. doi:10.1016/j.placenta.2009.02.009 19375795 PMC2697319

[14] Arnold DN , Brezzi F , Cockburn B , Donatella ML . Unified analysis of discontinuous Galerkin methods for elliptic problems. SIAM J Numer Anal. 2006;39:1749‐1779. doi:10.1137/S0036142901384162

[15] Giani S , Houston P . Goal‐oriented adaptive composite discontinuous Galerkin methods for incompressible flows. J Comput Appl Math. 2014;270:32‐42. doi:10.1016/j.cam.2014.03.007

[16] Paul H . AptoFEM FInite Element Analysis Software. 2024.

[17] Geuzaine C , Remacle JF . Gmsh: a 3‐D finite element mesh generator with built‐in pre‐ and post‐processing facilities. Int J Numer Methods Eng. 2009;79:1309‐1331. doi:10.1002/NME.2579

[18] Cockburn B , Kanschat G , Schötzau D . A locally conservative LDG method for the incompressible Navier‐stokes equations. Math Comput. 2005;74:1067‐1095. doi:10.1090/S0025-5718-04-01718-1

[19] Dellschaft NS , Hutchinson G , Shah S , et al. The haemodynamics of the human placenta in utero. PLoS Biol. 2020;18:e3000676. doi:10.1371/journal.pbio.3000676 32463837 PMC7255609

[20] Waluch V , Bradley WG . NMR even Echo Rephasing in slow laminar flow. J Comput Assist Tomogr. 1984;8:594‐598. doi:10.1097/00004728-198408000-00003 6330184

[21] Le Bihan D , Breton E , Lallemand D , Grenier P , Cabanis E , Laval‐Jeantet M . MR imaging of intravoxel incoherent motions: application to diffusion and perfusion in neurologic disorders. Radiology. 1986;161:401‐407. doi:10.1148/RADIOLOGY.161.2.3763909 3763909

[22] Lecarpentier E , Bhatt M , Bertin GI , Deloison B , Salomon LJ . Computational fluid dynamic simulations of maternal circulation: wall shear stress in the human placenta and its biological implications. PLoS One. 2016;11:e0147262. doi:10.1371/journal.pone.0147262 26815115 PMC4729471

[23] Serov AS , Salafia CM , Brownbill P , Grebenkov DS , Filoche M . Optimal villi density for maximal oxygen uptake in the human placenta. J Theor Biol. 2014;364:383‐396. doi:10.1016/j.jtbi.2014.09.022 25261730

[24] Shao X , Liu D , Martin T , et al. Measuring human placental blood flow with multidelay 3D GRASE pseudocontinuous arterial spin labeling at 3T. J Magn Reson Imaging. 2018;47:1667‐1676. doi:10.1002/JMRI.25893 29135072 PMC5951737

[25] Slator PJ , Hutter J , Palombo M , et al. Combined diffusion‐relaxometry MRI to identify dysfunction in the human placenta. J Cereb Blood Flow Metab. 2016;37:2987‐3000. doi:10.1177/0271678X16681310 30883915 PMC6519240

[26] Nelson SM , Coan PM , Burton GJ , Lindsay RS . Placental structure in type 1 DiabetesRelation to fetal insulin, leptin, and IGF‐I. Diabetes. 2009;58:2634‐2641. doi:10.2337/DB09-0739 19690062 PMC2768170

[27] Langhoff L , Grønbeck L , Von Huth S , et al. Placental growth during Normal pregnancy ‐ a magnetic resonance imaging study. Gynecol Obstet Invest. 2017;82:462‐467. doi:10.1159/000452661 27960180

[28] Kingdom JCP , Kaufmannb P . Oxygen and placental villous development: origins of fetal hypoxia. Placenta. 1997;18:613‐621. doi:10.1016/s0143-4004(97)90000 9364596

[29] Hutchinson G , Turbull A , Dewick L , et al. A combined T_2_* weighted gradient echo and 1D quantitative flow sequence for investigating placental function. Proceedings of the 33rd Annual Meeting of ISMRM, Singapore. The International Society for Magnetic Resonance in Medicine; 2024.

