# Supplementary material for: The effects of maternal flow on placental diffusion‐weighted MRI and intravoxel incoherent motion parameters
Source: Magn Reson Med. 2024 Nov 28;93(4):1629–41. doi: 10.1002/mrm.30379 (PMC11782734; doi:10.1002/mrm.30379)
Supplement: Supplementary file 1 — Figure S1. Figure of simulated placenta and placentone geometry. Figure S2. Figure of resistance of flow, flow direction and v′ for the simulated placenta. Figure S3. Figure unmasked v′ fit, showing all placental voxels. Figure S4. Figure showing additional example maps for a further healthy pregnancy and a compromised pregnancy. Figure S5. Figure showing individual subject data from the fIVIM parameter boxplots in Figure 4. Table S1. Table of dimensions of placental geometry and parameters associated with the blood flow model. Table S2. Table of discarded volumes per participant. Data S1. Detail of placental transition region. [file MRM-93-1629-s001.docx]

# Supporting material

## Figure S1


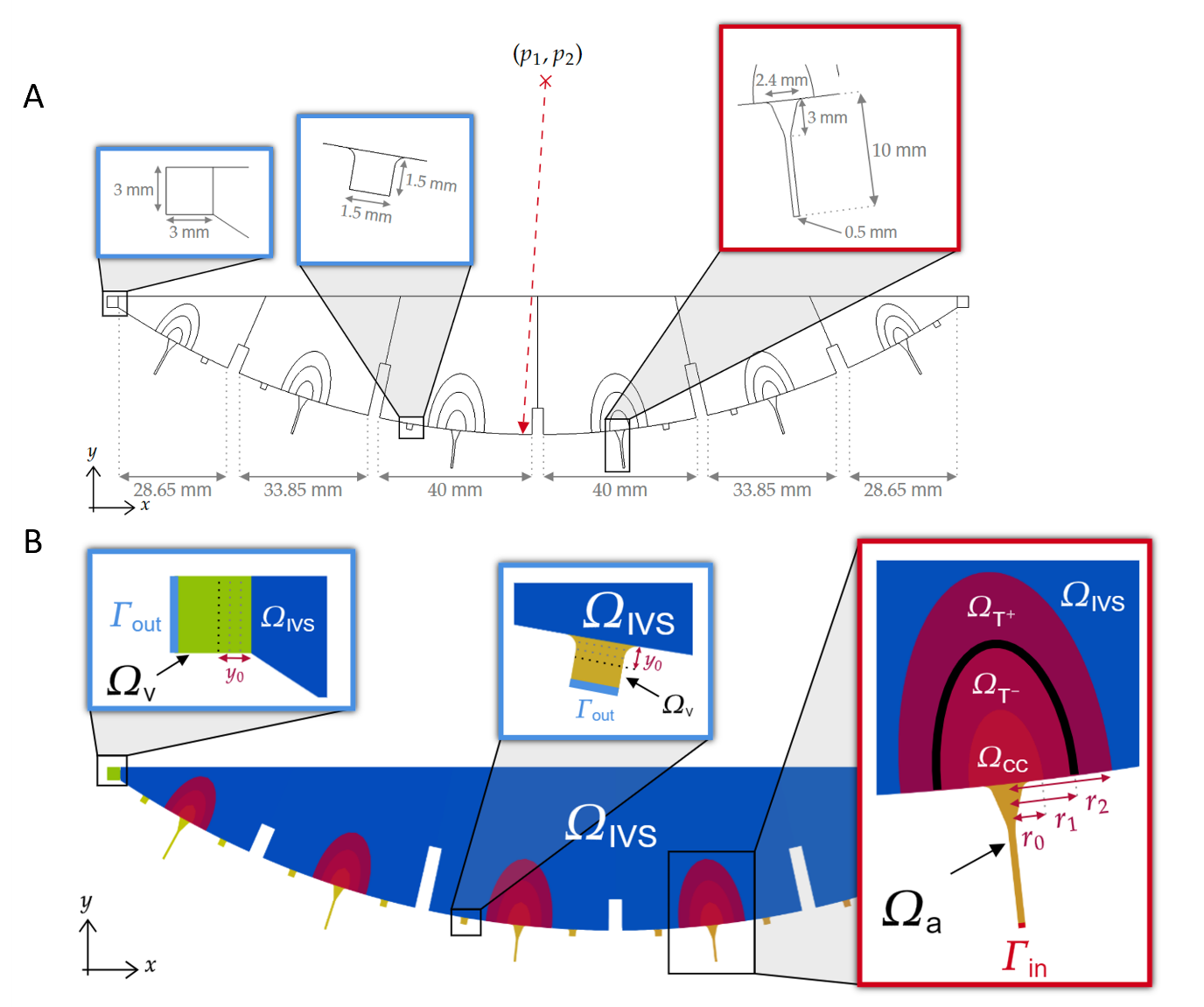


**Figure S1**: Illustrates the simulated 2D placenta geometry. (A) shows the dimensions associated with the placentones and vessels, and (B) shows how the arrangement of placentones within the domain. Notation is fully defined in A.1 but in brief, $\Omega_{\text{IVS}}$ is the region associated with the IVS, where villous tree material is present, whilst $\Omega_{\text{CC}}$ is the region associated with the central cavity, where no villous tree is present; $\Omega_{\text{T}^{-}}$ and $\Omega_{\text{T}^{+}}$ define an inner and outer transition region of villous tree material over the ellipses of radii $r_{0}$, $r_{1}$, and $r_{2}$. $\Omega_{\text{a}}$ is the region associated with the artery, where no villous tree is present. $\Omega_{\text{v}}$ is the region associated with the vein, where there is a transition in villous tree resistance over the distance $y_{0}$. $\Gamma_{\text{in}}$ and $\Gamma_{\text{out}}$ define the inflow and outflow edges, respectively. Not to scale.

## Figure S2


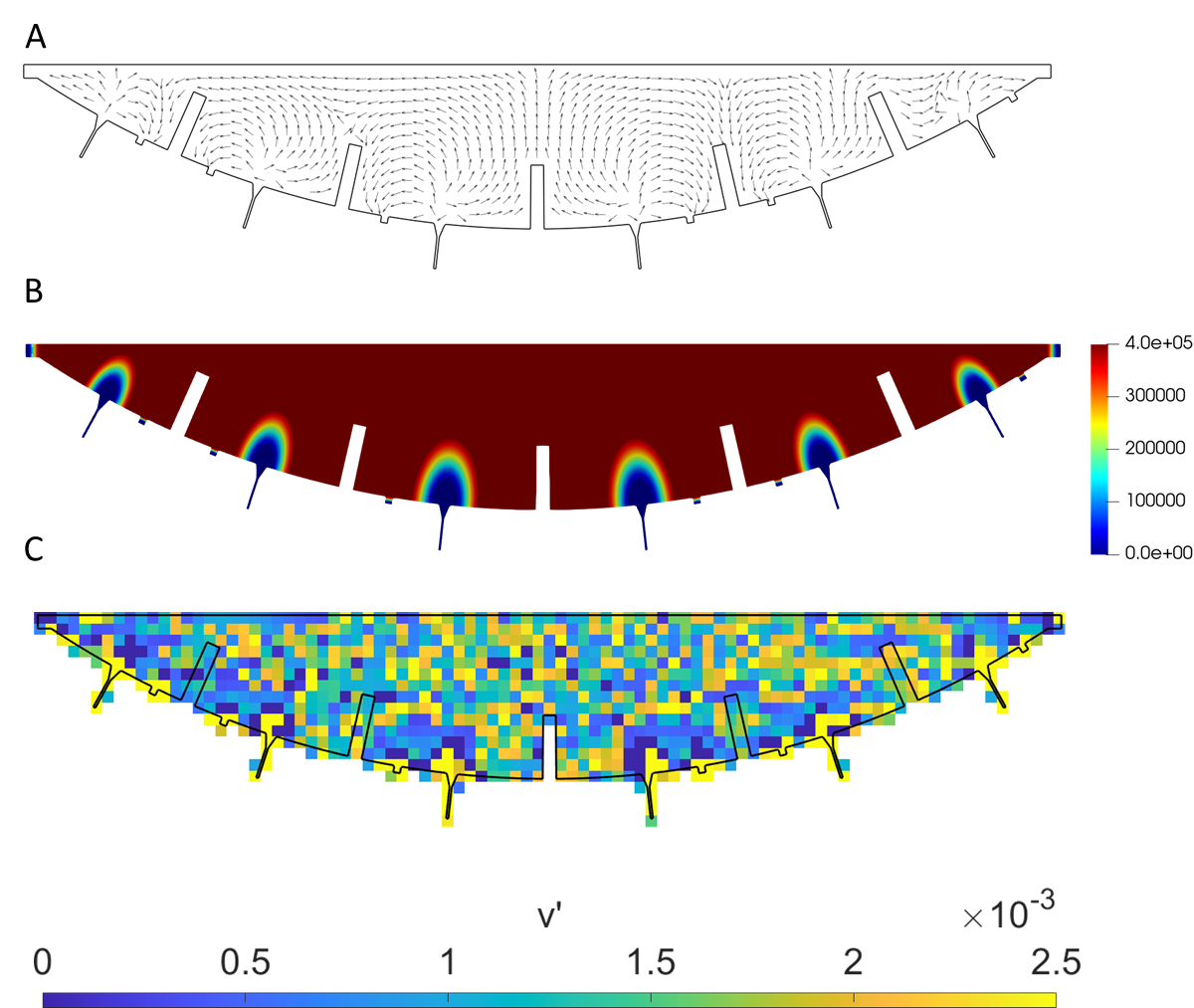


**Figure S2**: (A) The resistance to flow coefficient, $\Psi$. (B) An alternative visualisation of Figure 2B, where the arrows have equal length and simply show the direction of the mean flow over all isochromats in each pixel (C) The map of $v'$ from IVIM model fitted to the simulated data (this is the unmasked version of the map shown in Figure 3E).

## Figure S3


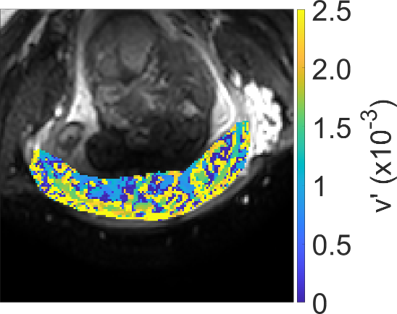


**Figure S3**: Full v’ map to be compared to the v’ figure in figure 4 which has been masked to only voxels where the rebound model outperformed the IVIM model according to an f test.

##
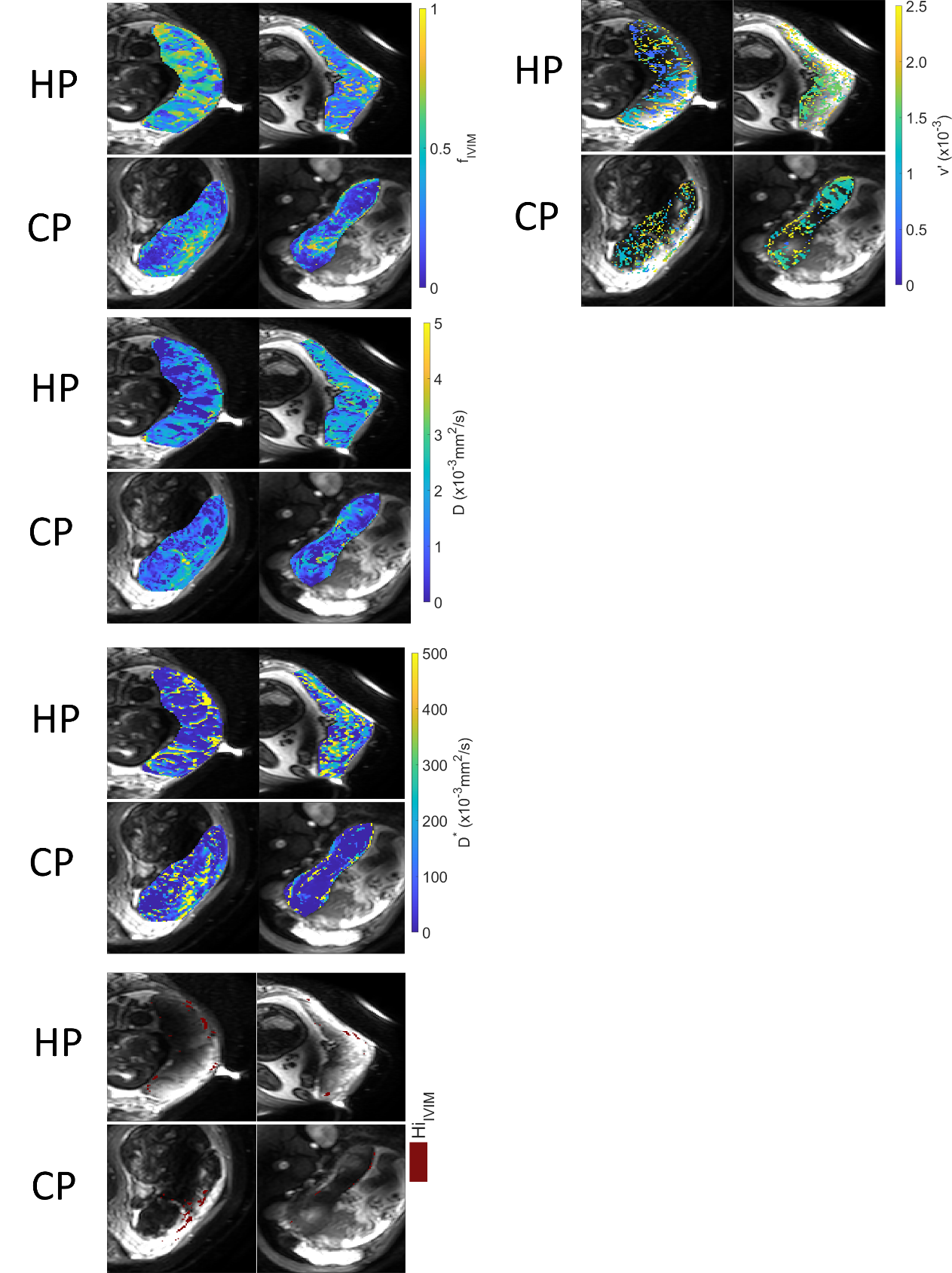
Figure S4

**Figure S4**: Further examples of parameter maps for fits to the data from a healthy pregnancy (HP01 and HP02, gestational ages 33+4 and 32+5 weeks) and Compromised pregnancies (CP 01 and CP02, gestational ages 35+4 and 32+1 weeks).

## Figure S5


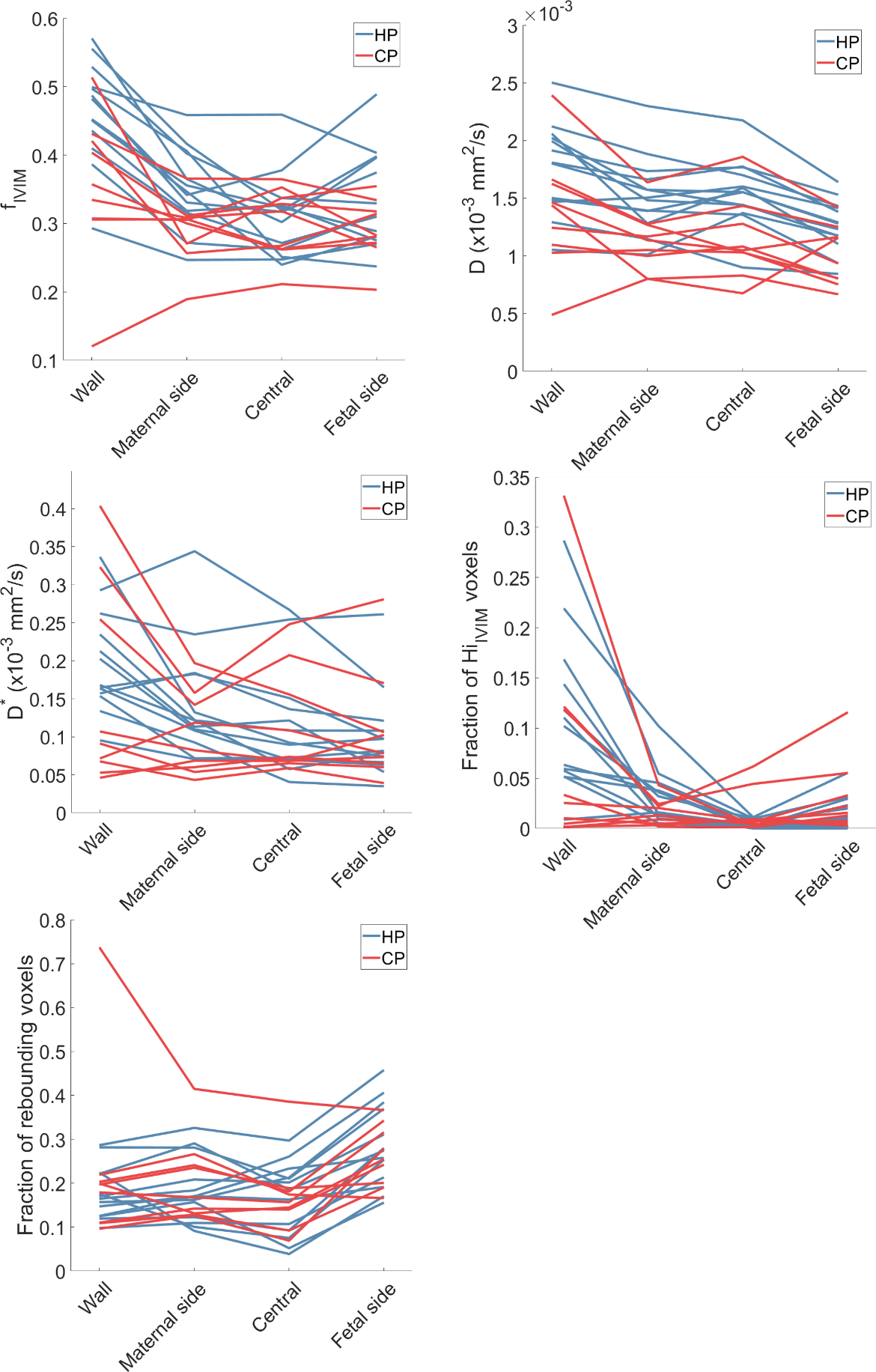


**Figure S5**: Results of the IVIM fit averaged over each ROI showing data for individual HP (blue) and CP (red) subjects to show trends across the 4 ROIs.

## Table S1

| Table S1 Dimensions of placental geometry and parameters associated with the blood flow model in Eq. 1. | | | |
| --- | --- | --- | --- |
| **Parameter** | **Value** | **Notes** | **Reference** |
| Artery width | $2.4 \text{mm}$ | Arteries begin at the base with a width of $0.5 \text{mm}$ ($2R$) and increase to $2.4 \text{mm}$ at $3 \text{mm}$ before meeting the central cavity. | ^1^ |
| Artery length | $10 \text{mm}$ | Distance from the basal plate where arterial boundary conditions are applied. | ^1^ |
| Basal plate vein width | $1.5 \text{mm}$ | — | — |
| Basal plate vein length | $1.5 \text{mm}$ | — | — |
| Marginal sinus vein width | $3 \text{mm}$ | — | — |
| Marginal sinus vein length | $3 \text{mm}$ | — | — |
| Central cavity width and height | $10 \text{mm}$, $20 \text{mm}$ | — | ^2,3^ |
| Vein $\Psi$ transition width | $1.2 \text{mm}$ | — | — |
| Central cavity $\Psi$ transition width | $4.8 \text{mm}$ | This is the transition tangential to the basal plate in the largest placentone; the normal transition here is $9.6 \text{mm}$; other cavity sizes are scaled proportionally to placentone width. | — |
| Vein transition centre $y_{1}$ | $1.2 \text{mm}$ | — | — |
| Cavity transition centre distance $a_{1}$ | $5 \text{mm}$ | This is the distance between the centre of an artery and the point at which the central ellipse meets the basal plate in the largest placentone; other cavity sizes are scaled proportionally to placentone width. | — |
| Placentone width | $40 \text{mm}$, $33.85 \text{mm}$, or $28.65 \text{mm}$ | The placentone widths decrease towards the periphery. | ^2,3^ |
| Placenta height | $36.26 \text{mm}$ | Calculated as a mean over several measured samples. | ^4^ |
| Placenta width | $220 \text{mm}$ | — | ^5^ |
| Wall height | $14.07 \text{mm}$ | Calculated as a mean over several measured samples. | ^6^ |
| Villous tree permeability $k$ | $1\times{10}^{-8} \text{m}^{\text{2}}$ | — | ^7,8^ |
| Dynamic viscosity $\mu$ | $4\times{10}^{-3} \text{Pa s}$ | — | ^7^ |
| Density $\rho$ | $10\times{10}^{3} \text{kg/}\text{m}^{\text{3}}$ | — | ^7^ |
| Artery blood speed (upstream) $V$ | $3.5\times{10}^{-1} \text{m/s}$ | Chosen such that $\left\vert\boldsymbol{v} \right\vert\approx0.1 \text{m/s}$ at the centre of the artery-IVS interface. | ^1,7,8^ |

**References**

1. Burton GJ, Woods AW, Jauniaux E, Kingdom JCP. Rheological and Physiological consequences of conversion of the maternal spiral arteries for uteroplacental blood flow during human pregnancy. Placenta. 2009;30(6):473-482. doi:10.1016/j.placenta.2009.02.009

2. Arnold DN, Brezzi F, Cockburn B, Donatella Marini L. Unified Analysis of Discontinuous Galerkin Methods for Elliptic Problems. https://doi.org/101137/S0036142901384162. 2006;39(5):1749-1779. doi:10.1137/S0036142901384162

3. Giani S, Houston P. Goal-oriented adaptive composite discontinuous Galerkin methods for incompressible flows. J Comput Appl Math. 2014;270:32-42. doi:10.1016/j.cam.2014.03.007

4. Afrakhteh M, Moeini A, Taheri MS, Haghighatkhah HR. Correlation between placental thickness in the second and third trimester and fetal weight. Rev Bras Ginecol Obstet. 2013;35(7):317-322. doi:10.1590/S0100-72032013000700006

5. Benirschke K, Burton GJ, Baergen RN. Pathology of the human placenta, sixth edition. Pathology of the Human Placenta. Published online January 1, 2012:1-941. doi:10.1007/978-3-642-23941-0/COVER

6. Amanitis D, Deligianni F, Abuhaimed R, Leach L. An ex-vivo macroscopic analysis of the human placental inter-lobular and inter-cotyledon septum and vessels therein from normal pregnancies. Placenta. 2023;140:e68-e69. doi:10.1016/J.PLACENTA.2023.07.218

7. Chernyavsky IL, Jensen OE, Leach L. A Mathematical Model of Intervillous Blood Flow in the Human Placentone. Placenta. 2010;31(1):44-52. doi:10.1016/J.PLACENTA.2009.11.003

8. Lecarpentier E, Bhatt M, Bertin GI, et al. Computational Fluid Dynamic Simulations of Maternal Circulation: Wall Shear Stress in the Human Placenta and Its Biological Implications. Published online 2016. doi:10.1371/journal.pone.0147262

## Table S2

| Table S2 list of discarded volumes from each participant | |
| --- | --- |
| **Participant ID** | **Discarded volumes (s/mm^2)** |
| HP01 |  |
| HP02 |  |
| HP03 |  |
| HP04 | 270,350,400 |
| HP05 | 147 |
| HP06 | 110,147,180,450 |
| HP07 |  |
| HP08 | 110,230,450 |
| HP09 |  |
| HP10 | 88,200 |
| HP11 | 3,270 |
| HP12 | 9,350 |
| HP13 | 32,88 |
| CP01 |  |
| CP02 | 400,450,500 |
| CP03 | 147,500 |
| CP04 |  |
| CP05 |  |
| CP06 | 1,32,300,450,500 |
| CP07 | 88 |
| CP08 |  |
| CP09 | 180,200,230 |

## Supplementary detail S1 Placental transition region

recall that the reaction coefficient in Eq. 1-2 varies spatially, similar to previous work^9^. The central cavity is defined by half an ellipse (a semi-ellipse) with a semi-minor axis of $a_{1}$ and a semi-major axis of $b_{1}$ where $2a_{1}=b_{1}$ with $a_{1}$ denoting the semi-axis of the ellipse that is tangential to the basal plate. We then add two more ellipses at equal distances inside and outside of the first ellipse, acting as a transition region. We introduce a smooth transition function defined as

$$\beta_{s_{0},s_{1},s_{2}}:=\left\{ \begin{aligned} 0, &\text{if }0 \leq s\leq s_{0}, \\ \frac{1}{2}\left[ \frac{\tanh(\gamma\left\{ \frac{s-s_{1}}{s_{2}-s_{1}} \right\})}{\tanh(\gamma)}+1 \right], &{\text{if }s}_{0}<s<s_{2}, \\ 1, &{\text{if }s}_{2}\leq s, \end{aligned} \right.$$

where $s_{2}-s_{0}$ gives the transition width, and we assume the relation $s_{1}\equiv\frac{s_{0}+s_{2}}{2}$. We note that $\beta$ differs from $\text{tanh}$, as $\text{tanh}$ has asymptotes at $\pm1$, whereas $\beta$ has no such asymptotes; $\beta$ attains maximum and minimum values by taking a ‘cut-off’ value, parameterised by $\gamma$, outside which $\beta$ attains its extrema. We note that the scaling $\gamma$ is included so that $\beta$ is continuous everywhere and is fixed $\gamma=0.999$.

The ellipses which form the geometry of the smooth transition regions at the boundary of the central cavity are defined by

$$r_{0}\left( \theta\right):=\frac{a_{0}b_{0}}{\sqrt{a_{0}^{2}\sin^{2} \left( \theta\right)+b_{0}^{2}\cos^{2} \left( \theta\right)}},$$

$$r_{1}\left( \theta\right):=\frac{a_{1}b_{1}}{\sqrt{a_{1}^{2}\sin^{2} \left( \theta\right)+b_{1}^{2}\cos^{2} \left( \theta\right)}},$$

$$r_{2}\left( \theta\right):=\frac{a_{2}b_{2}}{\sqrt{a_{2}^{2}\sin^{2} \left( \theta\right)+b_{2}^{2}\cos^{2} \left( \theta\right)}},$$

where $\boldsymbol{x}\equiv(x, y)$ are 2D Cartesian coordinates, and

$$\theta\left( \boldsymbol{x} \right):=\arctan\left( \frac{y-c_{2}}{x-c_{1}} \right),$$

$r\left( \boldsymbol{x} \right):=\sqrt{\left( \boldsymbol{x}-\boldsymbol{c} \right)\cdot\left( \boldsymbol{x}-\boldsymbol{c} \right)}$are 2D polar coordinates with $r=0$ at $\boldsymbol{c}\equiv(c_{1},c_{2})$ and $\theta=0$ tangential to the interface between $\Omega_{\text{CC}}$ and $\Omega_{\text{a}}$ (pointing in the anticlockwise direction). Here, $\boldsymbol{c}$ is the point at the centre of where an artery meets the placenta. The smooth transition regions in the veins are defined using

$$m\left( \boldsymbol{x} \right):=\left( \boldsymbol{x}-\boldsymbol{c} \right)\cdot\hat{\boldsymbol{n}},$$

where $\hat{\boldsymbol{n}}:=\boldsymbol{n}/\left| \boldsymbol{n} \right|$, $\boldsymbol{n=c}-\boldsymbol{p}$, and $\boldsymbol{p}\equiv(p_{1},p_{2})$ is the centre of the large circle that traces out the curve on the basal plate. Here, $\boldsymbol{c}$ is the point at the centre of where a vein meets the placenta.

The coefficient $\Psi$ is then given as

$$\Psi\left( \boldsymbol{x} \right):=\frac{\mu}{k}\left\{ \begin{aligned} 0, &\boldsymbol{x}\in\Omega_{\text{a}}\cup\Omega_{\text{CC}}, \\ \beta_{r_{0},r_{1},r_{2}}(r(\boldsymbol{x})), &\boldsymbol{x}\in\Omega_{\text{T}^{-}}\cup\Omega_{\text{T}^{+}}, \\ \beta_{y_{0},y_{1},y_{2}}(m(\boldsymbol{x})), &\boldsymbol{x}\in\Omega_{\text{v}}, \\ 1, &\boldsymbol{x}\in\Omega_{\text{IVS}}\text{,} \end{aligned} \right.$$

where the values of $\mu$ and $k$ are taken from Table 2, and $y_{2}-y_{0}$ is chosen to give a transition region in the veins with $y_{1}=\frac{y_{0}+y_{2}}{2}$. $\Psi(\boldsymbol{x})$ is illustrated on the simulated placenta geometry in Figure S2A.

Boundary conditions are applied to Eq.1-2 with parabolic inflow velocity profile, a natural outlet condition on outflow, and no slip elsewhere. To be explicit, we set

$$\boldsymbol{v}=-V\frac{R^{2}-r^{2}}{R^{2}}\boldsymbol{n}\text{ on }\Gamma_{\text{in}},$$

$$\mu\boldsymbol{\nabla}\boldsymbol{v\cdot n}=p\boldsymbol{I\cdot n} \text{on }\Gamma_{\text{out}},$$

$$\boldsymbol{v}=\boldsymbol{0}\text{on }\Gamma\setminus\left( \Gamma_{\text{in}}\cup\Gamma_{\text{out}} \right),$$

where, for each artery, $\boldsymbol{c}$ is the point at the centre of where an artery meets the placenta, $\boldsymbol{n=n(c)}$ is the outward-pointing unit normal at $\boldsymbol{c}$, $R$ is the artery radius at the base, $V$ is the speed at the centre of the artery base, and $r=r(\boldsymbol{x})$ is the distance from a point $\boldsymbol{x}$ to $\boldsymbol{c}$.

**References**

9. Mekler T, Plitman Mayo R, Weissmann J, Marom G. Impact of tissue porosity and asymmetry on the oxygen uptake of the human placenta: A numerical study. Placenta. 2022;129:15-22. doi:10.1016/J.PLACENTA.2022.09.008
